# Silver Nanoparticles on Cellulose Surfaces: Quantitative Measurements

**DOI:** 10.3390/nano9050780

**Published:** 2019-05-22

**Authors:** Ana Patrícia Carapeto, Ana Maria Ferraria, Ana Maria Botelho do Rego

**Affiliations:** 1 BioISI—Biosystems & Integrative Sciences Institute, Faculty of Sciences, University of Lisboa, 1749-016 Lisboa, Portugal; 2 Centro de Química-Física Molecular and IN-Institute for Nanosciences and Nanotechnologies and IBB-Institute for Bioengineering and Biosciences, Instituto Superior Técnico, Universidade de Lisboa, 1049-001 Lisboa, Portugal; amrego@tecnico.ulisboa.pt

**Keywords:** cellulose, silver, nanoparticles, thin films, AFM, QCM, XPS

## Abstract

In this work, cellulose films pre-activated with carbonyldiimidazole (CDI) and grafted with 1,6-hexanediamine, were decorated with silver nanoparticles (AgNPs). The generation of AgNPs was followed by quartz crystal microbalance (QCM). The obtained films were characterized by X-Ray Photoelectron Spectroscopy (XPS) and imaged by atomic force microscopy (AFM). XPS confirmed the synthesis in situ of AgNPs on the film attesting their oxidation state. The results from the three techniques were compared showing how sound the quantitative treatment of the results issued from these techniques can be. The main objective of this work is exactly to show that the quantitative exploration of the results of different characterization techniques can and should be practiced systematically instead of just comparing them qualitatively.

## 1. Introduction

The use of a large battery of techniques to characterize a given system is, recently, a common approach. Specifically, for the study of systems involving surface functionalization, several microscopies (atomic force microscopy, AFM; scanning electron microscopy, SEM) as well as mass measuring tools (quartz crystal microbalance, QCM) and surface-specific spectroscopic techniques such as X-ray photoelectron spectroscopy (XPS) are frequently used together. However, their quantitative use to test how their different probing range can enrich the obtained information is rare.

Here, an example dealing with the functionalization of cellulose films with metallic nanoparticles will be used to exemplify how quantitative these studies can be.

Developing methods for immobilizing metallic nanoparticles (NPs) onto different surfaces is a subject of fundamental interest, as nanometric structures enable the appearance of novel properties, absent in bulk or even in micrometric dimensions. Silver NPs have been increasingly used in various fields. These nanoparticles are being successfully used in cancer diagnosis and treatment [1,2]. Nano-silver systems also present several advantages that make them very interesting for use as antimicrobial agents. They possess a very high activity against a broad range of microbes and parasites [3,4] as well as biofilm inhibition in medical implants [5]. Furthermore, the incorporation of releasable silver NPs into polymeric nanofibers could be a promising technique to provide effective antibacterial action during wound healing [6].

**Scheme 1:**
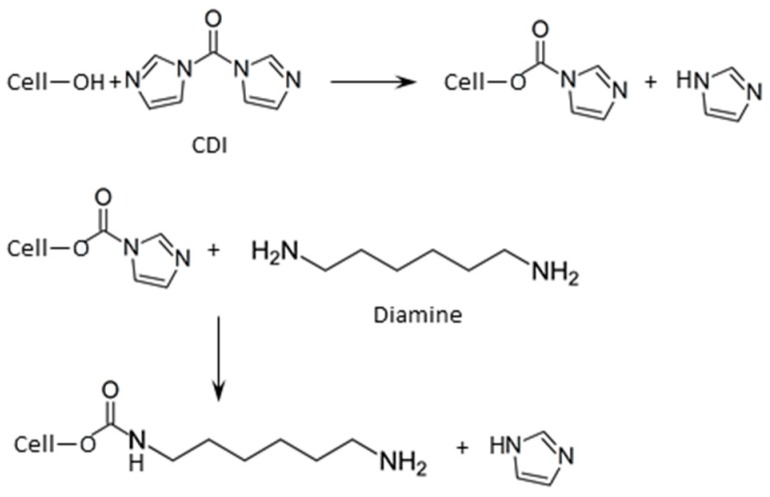
Activation of cellulose (Cell) with carbonyldiimidazole (CDI) followed by the functionalization with hexanediamine.

Natural cotton is one of the purest cellulose sources and is the most significant commercial source of natural fibers. As a material, cotton holds several attractive properties, including biocompatibility, biodegradability, and both thermal and chemical stability. Silver NPs linked to cellulose reportedly demonstrate antimicrobial activity through the release of nanoscale silver [7]. Preceding works published by our group on this subject, using cellulose in the form of films have shown that the nucleation and growth of silver NPs on the surface of cellulose films, interacting with a suitable silver salt solution, can be optimized by a prior chemical activation of the alcohol groups on the cellulose surface followed by the grafting of aliphatic diamine molecules (Scheme 1). One of the amine groups will be grafted on the film (specially to the cellulose primary alcohol) and the other one will complex silver ions. The growth of NPs is achieved by the interaction of aqueous dilute solutions of silver nitrate (AgNO_3_) with this modified cellulose film surface [8,9,10], the amine group coordinating to the silver ion and the cellulose also having a role in the partial reduction of further silver ions [11,12]. The procedure limits the generation of NPs only on the cellulose surface, keeping the dispersion medium completely exempt of them. The resulting NPs are, consequently, chemically immobilized on the cellulose surface.

The characterization of immobilized NPs at surfaces, usually includes the imaging by AFM. However, when trying to quantify the total amount of silver in the form of NPs, even if the image analysis allows for a proper evaluation of the NP size distribution, several assumptions need to be made, namely, concerning the shape of NPs. On the other hand, estimating the volume of nanoparticles immobilized on a surface using the QCM requires other assumptions, namely that the metal density in the NPs is the same as in the bulk, and that the silver composing the as-synthesized NP is in the metallic form. Such chemical characterization can be done by XPS. Since these three complementary techniques require different assumptions, the complementarity of the data is very important to validate the obtained results.

## 2. Materials and Methods

Acetone (puriss.) was obtained from Fluka (Steinheim, Germany). Absolute ethanol was from Riedel-de Haën (Steinheim, Germany). Dimethylsulfoxide (DMSO), hydrochloric acid (HCl, 37%), *N*,*N*′-Carbonyldiimidazole (≥90%) (CDI), 1,6-hexanediamine (98%) and silver nitrate (AgNO_3_, 99.9999%), were purchased from Aldrich (Steinheim, Germany). Deionized water (DIW) with a resistivity of 18.2 MΩ cm was supplied by a MILLIPORE system (Darmstadt, Germany), fed with distilled water.

QCM crystals (5 MHz Au/Cr, Stanford Research Systems, Sunnyvale, CA, USA) were degreased with acetone and ethanol, and dried under an argon flux. The cellulose films were produced by spin-coating (speed = 2000 rpm, *t* = 60 s, acceleration = 800 rpm/s) the clean crystal of Quartz/Au (electrode) with a trimethylsilylcellulose (TMSC) film from a solution in toluene (prepared as described in [13]) and exposing it immediately to a saturated HCl atmosphere to regenerate the cellulose. The film surface was then activated with CDI (QCM crystal/cellulose film was immersed in a CDI solution 5 × 10^−2^ M in DMSO, for 2 h), functionalized with 1,6-hexanediamine (QCM crystal/activated cellulose film was immersed in a diamine solution 1 × 10^−2^ M in DMSO, for 2 h) and thoroughly rinsed with ethanol and water to remove any physisorbed amine.

The quartz crystal microbalance used in this work was the QCM 200 from Stanford Research Systems. The QCM crystal coated with the cellulose film (grafted with 1,6-hexanediamine), was mounted in the QCM and placed in interaction with AgNO_3_ (5 × 10^−3^ M aqueous solution) by injecting in the reaction chamber, perpendicularly to the film surface, a flow of the silver salt solution for 15 min and at room temperature. The variation in frequency (Δ*f*) was monitored as a function of the time. The Sauerbrey equation:

Δ*f* = −C_f_Δ*m*, (1)

where Δ*f* is the frequency change, Δ*m* is the mass change per unit area and C_f_ is the sensitivity factor for the crystal which is 56.6 Hz µg^−1^ cm^2^, relates the mass change per unit area at the QCM electrode surface to the observed change in the oscillation frequency of the crystal, provided that |Δ*f*|/*f*_0_ (where *f*_0_ = 5 MHz is the resonant frequency of the fundamental mode of the unloaded crystal) is less than 2%. It was used to obtain the mass gain as the nanoparticles grew on the cellulose surface [14].

The resulting functionalized films were imaged by AFM (D3100 with a Nanoscope IIIa controller from Digital Instruments, Santa Barbara, CA, USA) carried out in tapping mode under ambient conditions with TESP tips (Material: 0.01–0.025 Ω cm Antimony (n) doped Si; *R*: 3.25–4.75 µm; *L*: 110–140 µm; *W*: 30–50 µm; *f*_0_: 279–389 kHz; *k*: 20–80 N/m; estimated nominal radius = 8 nm), Bruker AFM Probes (Camarillo, CA, USA). The AFM images were analyzed with the software WSxM (5.0 Develop 6.5, Nanotec Electronica S.L., Madrid, Spain) [15]. However, the number and volume of the nanoparticles could not be estimated by using the WSxM flooding tool, which allows to detect different features of the probed area, like hills and/or holes. In this case, and due to the roughness of the polymeric film and the dimensions of the nanoparticles, when detecting hills above a given height, besides the nanoobjects identified as silver nanoparticles, it also detects cellulosic bundles whose area and volume are also quantified by the software. To avoid misleading measurements, a visual inspection of the images was done, counting and measuring the sizes of the nearly spherical nanoparticles existing at the surface. The reliability of such simplistic data collection was analyzed by comparing the results with other data obtained by a very different technique of analysis, the QCM.

The hybrid film was characterized by XPS, using a XSAM 800 dual anode spectrometer from KRATOS (Manchester, UK). Non-monochromatic Al Kα X-rays (hν = 1486.6 eV) were used and the take-off angle was 45°. The analyzed area in high magnification mode is approximately 1 mm × 3 mm. Other operation and acquisition conditions and data treatment details were as described in [16]. No charge correction was needed. The quantification factors were 0.25, 0.66, 0.42, and 5.2 for C 1s, O 1s, N 1s, and Ag 3d, respectively.

## 3. Results

The growth of NPs was followed by QCM and the QCM crystal with the hybrid film was imaged by AFM. The chemical characterization was performed by XPS.

### 3.1. QCM

**Figure 1:**
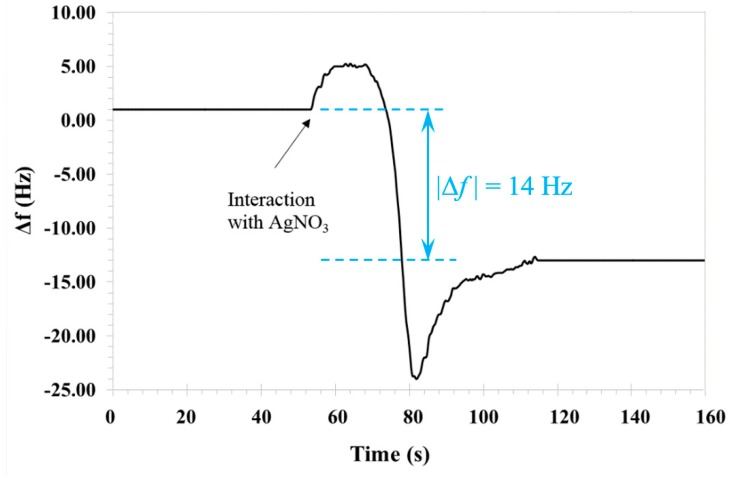
Quartz crystal microbalance (QCM) result: variation of the crystal frequency (∆*f*) after interaction of the cellulose film (grafted with 1,6-hexanediamine) with AgNO_3_ monitored as a function of the time.

The NPs growth was followed by QCM (Figure 1). From QCM measurements, |Δ*f*| is less than 20 Hz, which for a crystal resonant frequency of 5 MHz, corresponds to a |Δ*f*|/*f*_0_ less than 4 × 10^−4^%, largely within the Sauerbrey Equation validity range (|Δ*f*|/*f*_0_ < 2%). The mass of nanoparticles formed on the film surface is, therefore, computed from the Sauerbrey Equation (1), 0.22 ± 0.04 µg/cm^2^. This value was obtained based on three independent measurements (three samples) ranging from |Δ*f*| = 11 to 14 Hz, yielding an average value of 12.5 ± 2.1 Hz. Assuming the same density for silver NPs as for bulk silver (ρ = 10.5 g/cm^3^), the NPs should have a total volume of 210,331 ± 35,694 nm^3^/μm^2^. Even if the assumption about the density is approximate, the order of magnitude should be this one.

### 3.2. AFM

**Figure 2:**
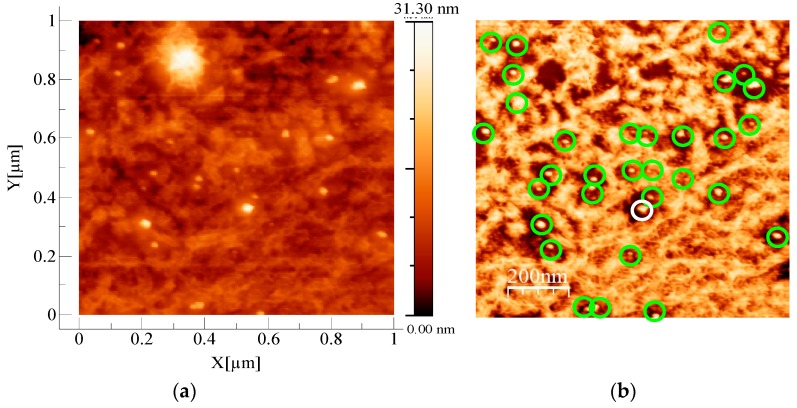

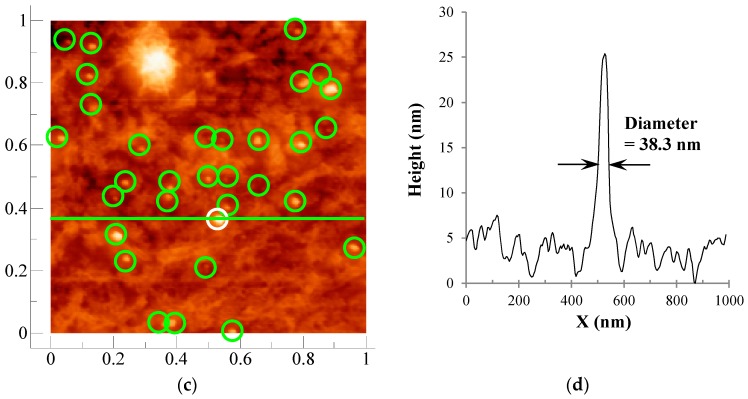
(**a**) Atomic force microscopy (AFM) image of hybrid cellulose film (1000 nm × 1000 nm) in topographic mode; (**b**) AFM image of hybrid cellulose film, the same as in (**a**) in phase mode; circles were drawn around the undoubtedly silver nanoparticles; (**c**) AFM image of (**a**) superposed with the same circles which were drawn in the phase image in (**b**); (**d**) Height profile for a horizontal line crossing the white circle center.

Cellulose ultrathin hybrid film was imaged by AFM. Each measurement consisted of three images of the surface, 1 × 1 μm^2^ (or lower): height, amplitude, and phase images. The images presented in Figure 2 correspond to one of seven different points of the surface. Topographic and phase images are shown in Figure 2a,b respectively. Discrete NP are observed after the interaction with AgNO_3_. They are better identified in the phase image than in the topographic one. Since the cellulose film surface is very rough, no automatic tool (such as WSxM) can be applied to the image to count and measure the nanoparticles.

To make an estimation of the nanoparticle volume, a “manual” method was adopted: the NPs were visually identified in the phase image and encircled (Figure 2b).

This set of circles was grouped and paste on the topographic image (Figure 2c). Lines parallel to the x axis were drawn crossing the center of the encircled particle and the respective profile was drawn automatically by the software. The full width at half maximum of the profile feature was measured and taken as the particle diameter (see Figure 2d for an example). Also the heights were measured and taken as the radius of the sphere.

With this procedure, 32 NPs could be counted and the total volume, *∑_i_* 4/3*π(D_i_* /2*)*^3^, was computed to be 225,000 nm^3^. Using the heights, the volume obtained, *∑_i_* 4/3π*h_i_*^3^, was 199,000 nm^3^. The associated error is hard to estimate, given the intrinsic nature of the measurements in AFM and the made assumptions. Anyway, the relative difference for the average value found by the QCM method, is less than 7%.

### 3.3. XPS

**Figure 3:**
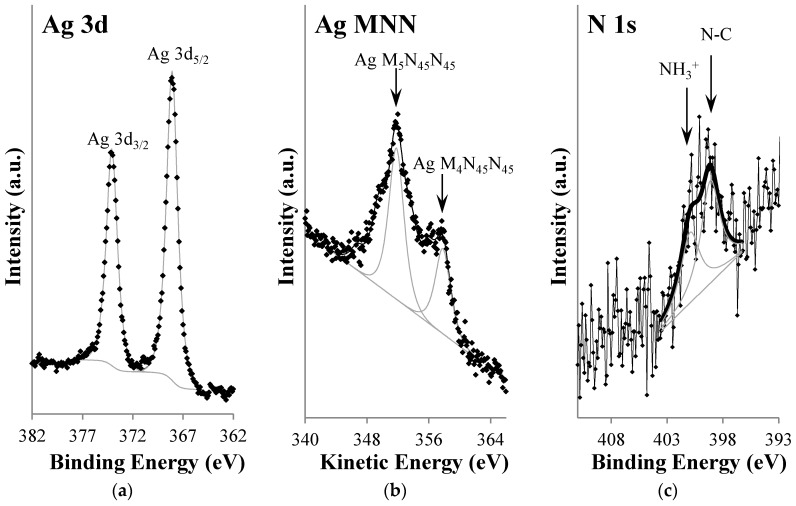
X-ray photoelectron spectroscopy (XPS) regions: (**a**) Ag 3d; (**b**) Ag MNN; and (**c**) N 1s.

The chemical characterization was performed by XPS (Figure 3). XPS results attest undoubtedly that the Ag 3d_5/2_ peak, centered at 368.1 ± 0.1 eV corresponds to metallic silver (Ag^0^) (Figure 3a). The silver oxidation state was deduced from the Auger parameters equal to 725.9 ± 0.1 eV and 719.9 ± 0.1 eV for the pairs [Binding Energy (BE) (Ag 3d_5/2_), Kinetic Energy (KE) (Ag M_4_N_45_N_45_)], and [Ag 3d_5/2_, Ag M_5_N_45_N_45_], respectively [17]. It is worth noting that for Ag 3d photoelectrons (kinetic energy ≈ 1118 eV), the attenuation length, λ, in silver is 1.6 nm [18] which gives a probe depth, *d*, taking *d* = 5λ (99.32% of the signal comes from this depth), of ~8.0 nm. Therefore, for particles with radii larger than 8 nm, this information does not come from the inner part of the particle but rather from an outer shell 8 nm thick.

N 1s shows that no unreacted NO_3_^−^, from the silver precursor, remains in the film, since it would be detected around 407 eV (Figure 3c). The nitrogen detected at 399.1 ± 0.2 eV comes from amine C–NH_2_ groups and carbamate groups C–N=O, formed upon cellulose activation with CDI, and, at higher binding energy (400.9 ± 0.2 eV), from protonated –NH_3_^+^ groups. Other diamine, cellulose and gold (substrate) features are detected in C 1s, O 1s, and Au 4f XPS regions (not shown). The fact that gold is detected confirms how thin the cellulose films here studied are. XPS analysis depth is of the order of 10 nm. Therefore, films are, in average, thinner than that value, confirming thicknesses estimated earlier for similar films [16].

## 4. Discussion and Conclusions

The values obtained with AFM and QCM are presented in Table 1. Given the rough approximations used in both estimations, and the consequent associated error, the two values which differ from each other less than 7%, may be considered the same.

This means that: (i) the used approximations are not too crude and the method can be soundly applied for other systems and (ii) the nanoparticles were really formed at the film extreme surface as expected given the low thickness of the films stated above and its functionalization with the diamines.

One can, then, conclude that the AgNPs synthesis procedure here described limits the generation of NPs only to the cellulose surface, keeping the dispersion medium (solution) completely exempt of them. The resulting NPs are, consequently, chemically immobilized on the cellulose surface.

The values obtained for the nanoparticles volume using AFM and QCM techniques are, within the experimental error, exactly the same in spite of the different sizes of the explored sample zones (of the order of a few cm^2^ in QCM and of µm^2^ in AFM). XPS is not adequate for estimating the NP volume but confirms that the silver oxidation state is Ag^0^. This information is indispensable to make the estimations based on AFM and QCM results. This work shows that data obtained through the combined use of these analysis techniques allow for sound and reliable estimations of volume NPs, provided that they are essentially formed on the extreme surface of materials and should be used in that way more often. This demonstration of the quantitative use of these techniques opens the way to further exploration of QCM and AFM (or SEM, depending on the involved sizes) data when properly combined with XPS.

## Figures and Tables

**Table 1:** Atomic force microscopy (AFM) and quartz crystal microbalance (QCM) values for the calculation of Ag nanoparticles volume.

AFM			QCM	
Number of NPs	32		Δ*f*	−12.5 ± 2.1 Hz
	Computed from measured diameters	Computed from measured heights	∆*m*	0.22 ± 0.04 µg/cm^2^
Ag NPs volume/Area	225,000 nm^3^/µm^2^	199,000 nm^3^/µm^2^	210,331 ± 35,694 nm^3^/µm^2^	
